# The genome sequence of a snakefly, *Xanthostigma xanthostigma *(Schummel, 1832)

**DOI:** 10.12688/wellcomeopenres.23674.1

**Published:** 2025-02-07

**Authors:** Steven Falk, Liam M. Crowley, Maxwell V. L. Barclay, Emma Taluy, University of Oxford Genome Acquisition Lab, Natural History Museum Genome Acquisition Lab, Darwin Tree of Life Barcoding collective, Wellcome Sanger Institute Tree of Life Management, Samples and Laboratory team, Wellcome Sanger Institute Scientific Operations: Sequencing Operations, Wellcome Sanger Institute Tree of Life Core Informatics team, Tree of Life Core Informatics collective, Darwin Tree of Life Consortium

**Affiliations:** 1 Independent researcher, Kenilworth, Warwickshire, England, UK; 2 University of Oxford, Oxford, England, UK; 3 Natural History Museum, London, England, UK; 4 Wellcome Sanger Institute, Hinxton, England, UK

**Keywords:** Xanthostigma xanthostigma, snakefly, genome sequence, chromosomal, Raphidioptera

## Abstract

We present a genome assembly from an individual male snakefly, *Xanthostigma xanthostigma* (Arthropoda; Insecta; Raphidioptera; Raphidiidae). The genome sequence has a total length of 623.30 megabases. Most of the assembly (99.74%) is scaffolded into 13 chromosomal pseudomolecules, including the X sex chromosome. The mitochondrial genome has also been assembled and is 17.75 kilobases in length. Gene annotation of this assembly on Ensembl identified 13,251 protein-coding genes.

## Species taxonomy

Eukaryota; Opisthokonta; Metazoa; Eumetazoa; Bilateria; Protostomia; Ecdysozoa; Panarthropoda; Arthropoda; Mandibulata; Pancrustacea; Hexapoda; Insecta; Dicondylia; Pterygota; Neoptera; Endopterygota; Neuropterida; Raphidioptera; Raphidiidae; *Xanthostigma*; *Xanthostigma xanthostigma* (Schummel, 1832) (NCBI:txid887762)

## Background

*Xanthostigma xanthostigma* is a member of the Raphidiidae family of snake flies (Raphidoptera); a poorly documented group that is challenging to observe in the wild ( Pendleton & Pendleton, 2024a; Pendleton & Pendleton, 2024b). Raphidoptera primarily inhabit tree-tops; however, they are occasionally seen on the bark of tree trunks, especially on oaks and pines ( Friedrich, 2024; Pendleton & Pendleton, 2024a; Pendleton & Pendleton, 2024b). *X. xanthotigma* is widely distributed across the UK and frequently sighted in England, from London to Bristol, across East Anglia, Lincolnshire, Yorkshire, and notably Leicester ( Pendleton & Pendleton, 2024a). Additionally, there have been a few recordings in Nottinghamshire, including Sherwood Forest Country Park, Nettleworth Manor and Treswell wood ( Pendleton & Pendleton, 2024a; Pendleton & Pendleton, 2024b). In northern England, records are primarily from Cumbria and the North-East near County Durham ( Pendleton & Pendleton, 2024a).

Distinguishing *X. xanthostigma* from other Raphidiidae can be difficult, but the species exhibits several distinguishing features. Its head shape, which narrows posteriorly when viewed from above, along with coarse punctuations on the head and unique net-like patterns of wing venation, facilitate identification ( Pendleton & Pendleton, 2024a; Wolf *et al.*, 2024). Their pale yellow pterostigma, the specific vein counts running through it and the veins connecting the costa and sub-costa are additional identifying markers ( Friedrich, 2024; Haring *et al.*, 2011; Pendleton & Pendleton, 2024a). Females are differentiated from males by their long ovipositors, which they use to deposit their eggs in tree bark crevices ( Pendleton & Pendleton, 2024a; Pendleton & Pendleton, 2024b; Wolf *et al.*, 2024). The larvae take two years to mature, feeding on beetle larvae ( Aspöck, 2002). However, they are not active hunters and can be sluggish. The larvae undergo pupation underneath leaf litter or loose tree bark ( Pendleton & Pendleton, 2024a). Snake flies are most frequently recorded when they are newly emerging from their pupae between early May and June and can be found low on tree trunks or fence posts during this stage ( Friedrich, 2024; Pendleton & Pendleton, 2024a; Pendleton & Pendleton, 2024b). Interestingly, snake flies have maintained their distinct appearance since the Mesozoic era and possibly since they first emerged over 300 million years ago, making them excellent “living fossils” ( Aspöck, 2002; Haring *et al.*, 2011; Wolf *et al.*, 2024).

We present the genome assembly of a male *X. xanthostigma*. Understanding its genome could increase awareness of this poorly recorded species, providing deeper insights into these “living fossils” and how they have maintained their distinctive features over time ( Aspöck, 2002; Wolf *et al.*, 2024). We aim to facilitate further genomic research and advance understanding of the species’ evolutionary traits and ecological adaptations.

## Genome sequence report

**Figure 1.**
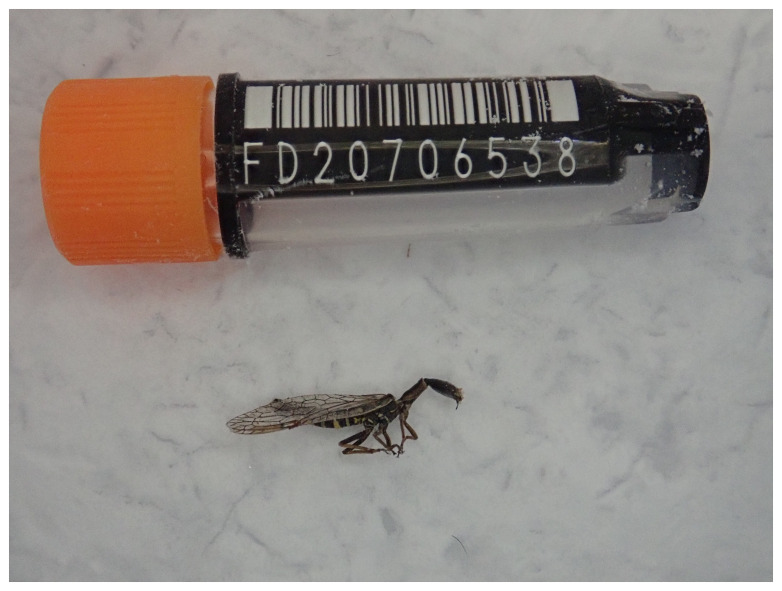
Photograph of the *Xanthostigma xanthostigma* (irXanXant1) specimen used for genome sequencing.

The genome of *Xanthostigma xanthostigma* ( Figure 1) was sequenced using Pacific Biosciences single-molecule HiFi long reads, generating a total of 18.51 Gb (gigabases) from 1.64 million reads, providing an estimated 29-fold coverage. Primary assembly contigs were scaffolded with chromosome conformation Hi-C data, which produced 89.81 Gb from 594.75 million reads. Specimen and sequencing details are summarised in Table 1.

**Table 1.** Specimen and sequencing data for *Xanthostigma xanthostigma*.

Project information			
**Study title**	Xanthostigma xanthostigma		
**Umbrella BioProject**	PRJEB66774		
**Species**	*Xanthostigma xanthostigma*		
**BioSample**	SAMEA10167082		
**NCBI taxonomy ID**	887762		
Specimen information			
**Technology**	**ToLID**	**BioSample accession**	**Organism part**
**PacBio long read sequencing**	irXanXant1	SAMEA10201330	Whole organism
**Hi-C sequencing**	irXanXant3	SAMEA112975698	Whole organism
Sequencing information			
**Platform**	**Run accession**	**Read count**	**Base count (Gb)**
**Hi-C Illumina NovaSeq 6000**	ERR12102446	5.95e+08	89.81
**PacBio Sequel IIe**	ERR12102464	1.64e+06	18.51

Assembly errors were corrected by manual curation, including 67 missing joins or mis-joins and 13 haplotypic duplications. This reduced the assembly length by 1.29% and the scaffold number by 34.09%, while the scaffold N50 increased by 4.45%. The final assembly has a total length of 623.30 Mb in 28 sequence scaffolds, with 58 gaps, and a scaffold N50 of 53.1 Mb ( Table 2).

**Table 2.** Genome assembly data for *Xanthostigma xanthostigma*, irXanXant1.1.

Genome assembly		
Assembly name	irXanXant1.1	
Assembly accession	GCA_963575645.1	
*Accession of alternate haplotype*	*GCA_963575605.1*	
Span (Mb)	623.30	
Number of contigs	87	
Number of scaffolds	28	
Longest scaffold (Mb)	84.21	
Assembly metrics *		*Benchmark*
Contig N50 length (Mb)	18.0	*≥ 1 Mb*
Scaffold N50 length (Mb)	53.1	*= chromosome N50*
Consensus quality (QV)	66.1	*≥ 40*
*k*-mer completeness	Primary assembly: 76.54%; alternate haplotype: 72.95%; combined assemblies: 98.88%	*≥ 95%*
BUSCO **	C:97.9%[S:96.8%,D:1.1%], F:0.9%,M:1.2%,n:2,124	*S > 90%, D < 5%*
Percentage of assembly mapped to chromosomes	99.74%	*≥ 90%*
Sex chromosomes	X	*localised homologous pairs*
Organelles	Mitochondrial genome: 17.75 kb	*complete single alleles*
Genome annotation of assembly GCA_963575645.1 at Ensembl		
Number of protein-coding genes	13,251	
Number of non-coding genes	500	
Number of gene transcripts	18,622	

* Assembly metric benchmarks are adapted from Rhie *et al.* (2021) and the Earth BioGenome Project Report on Assembly Standards September 2024. ** BUSCO scores based on the endopterygota_odb10 BUSCO set using version 5.4.3. C = complete [S = single copy, D = duplicated], F = fragmented, M = missing, n = number of orthologues in comparison. A full set of BUSCO scores is available at https://blobtoolkit.genomehubs.org/view/Xanthostigma_xanthostigma/dataset/GCA_963575645.1/busco.

**Figure 2.**
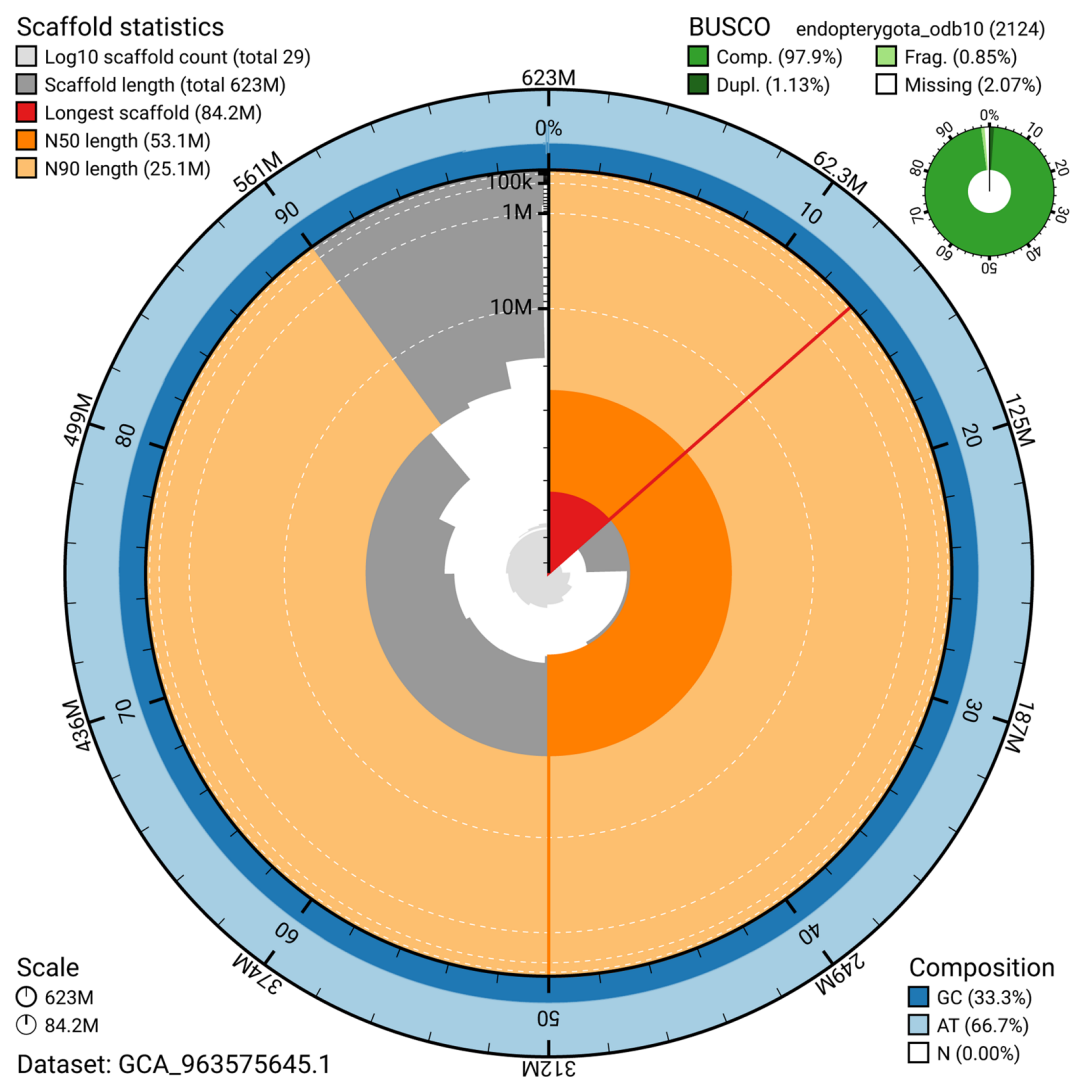
Genome assembly of *Xanthostigma xanthostigma*, irXanXant1.1: metrics. The BlobToolKit snail plot provides an overview of assembly metrics and BUSCO gene completeness. The circumference represents the length of the whole genome sequence, and the main plot is divided into 1,000 bins around the circumference. The outermost blue tracks display the distribution of GC, AT, and N percentages across the bins. Scaffolds are arranged clockwise from longest to shortest and are depicted in dark grey. The longest scaffold is indicated by the red arc, and the deeper orange and pale orange arcs represent the N50 and N90 lengths. A light grey spiral at the centre shows the cumulative scaffold count on a logarithmic scale. A summary of complete, fragmented, duplicated, and missing BUSCO genes in the endopterygota_odb10 set is presented at the top right. An interactive version of this figure is available at https://blobtoolkit.genomehubs.org/view/GCA_963575645.1/dataset/GCA_963575645.1/snail.

**Figure 3.**
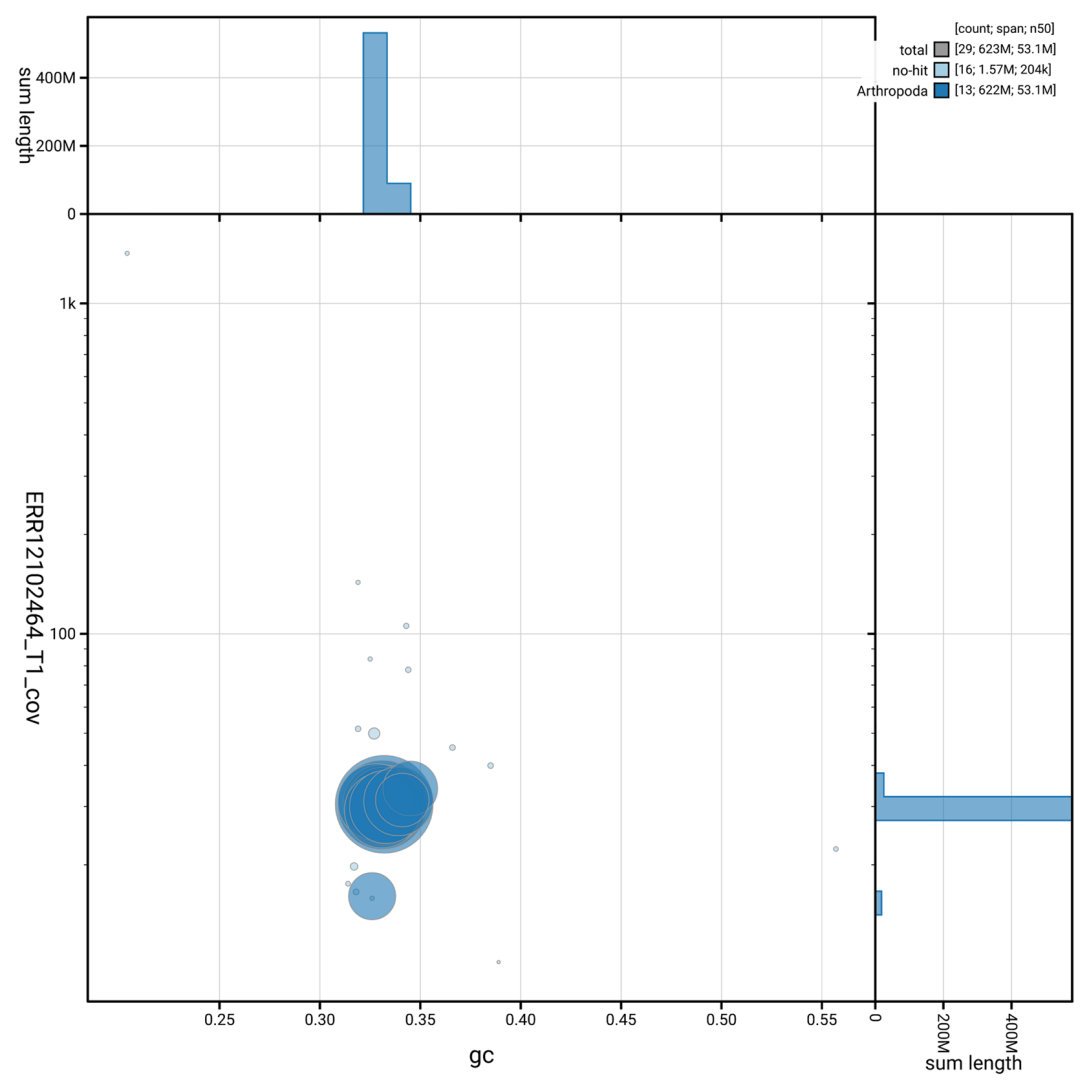
Genome assembly of *Xanthostigma xanthostigma*, irXanXant1.1: BlobToolKit GC-coverage plot showing sequence coverage (vertical axis) and GC content (horizontal axis). The circles represent scaffolds, with the size proportional to scaffold length and the colour representing phylum membership. The histograms along the axes display the total length of sequences distributed across different levels of coverage and GC content. An interactive version of this figure is available at https://blobtoolkit.genomehubs.org/view/GCA_963575645.1/dataset/GCA_963575645.1/blob.

**Figure 4.**
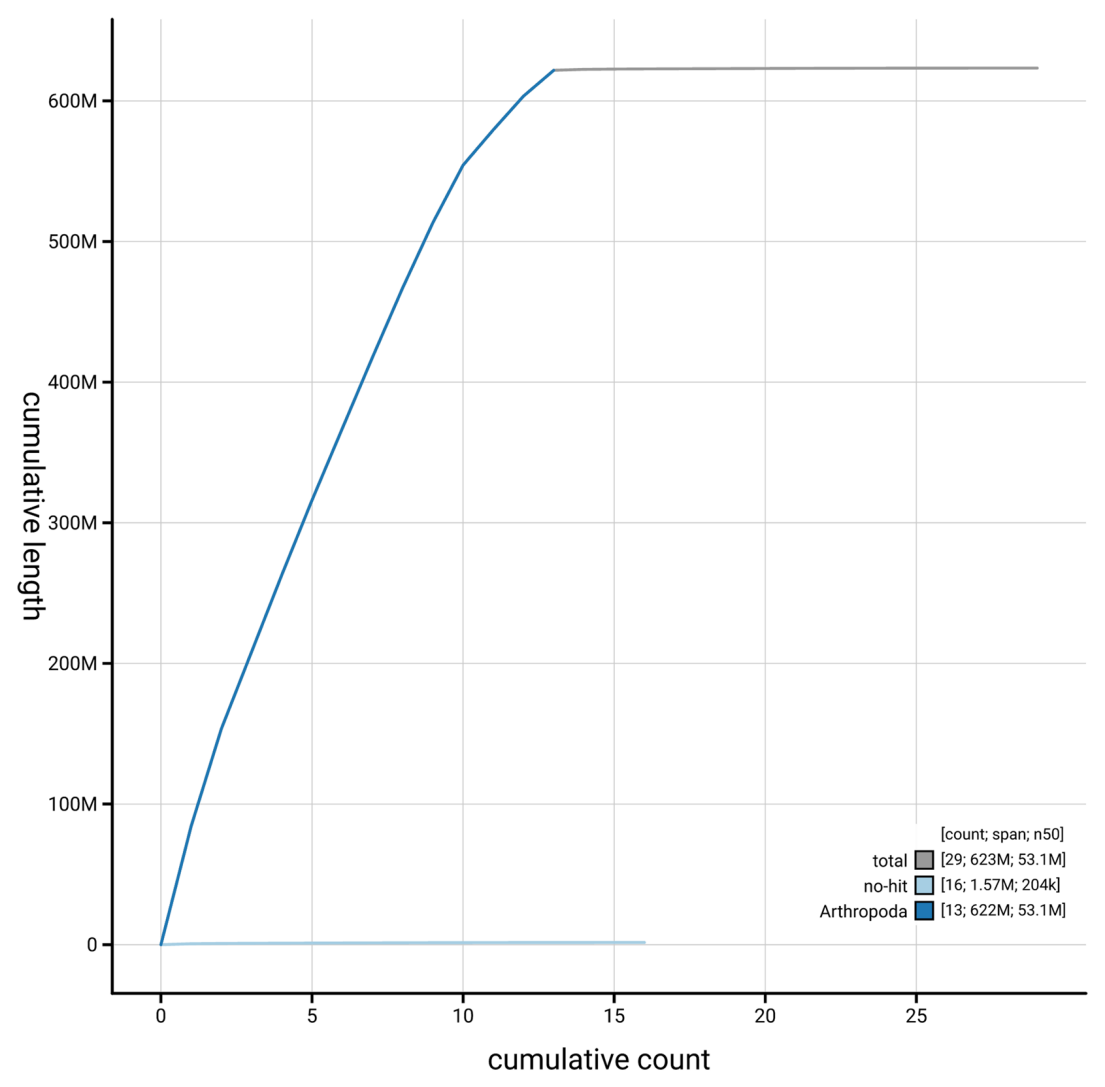
Genome assembly of *Xanthostigma xanthostigma* irXanXant1.1: BlobToolKit cumulative sequence plot. The grey line shows cumulative length for all scaffolds. Coloured lines show cumulative lengths of scaffolds assigned to each phylum using the buscogenes taxrule. An interactive version of this figure is available at https://blobtoolkit.genomehubs.org/view/GCA_963575645.1/dataset/GCA_963575645.1/cumulative.

The snail plot in Figure 2 provides a summary of the assembly statistics, indicating the distribution of scaffold lengths and other assembly metrics. Figure 3 shows the distribution of scaffolds by GC proportion and coverage. Figure 4 presents a cumulative assembly plot, with separate curves representing different scaffold subsets assigned to various phyla, illustrating the completeness of the assembly.

**Figure 5.**
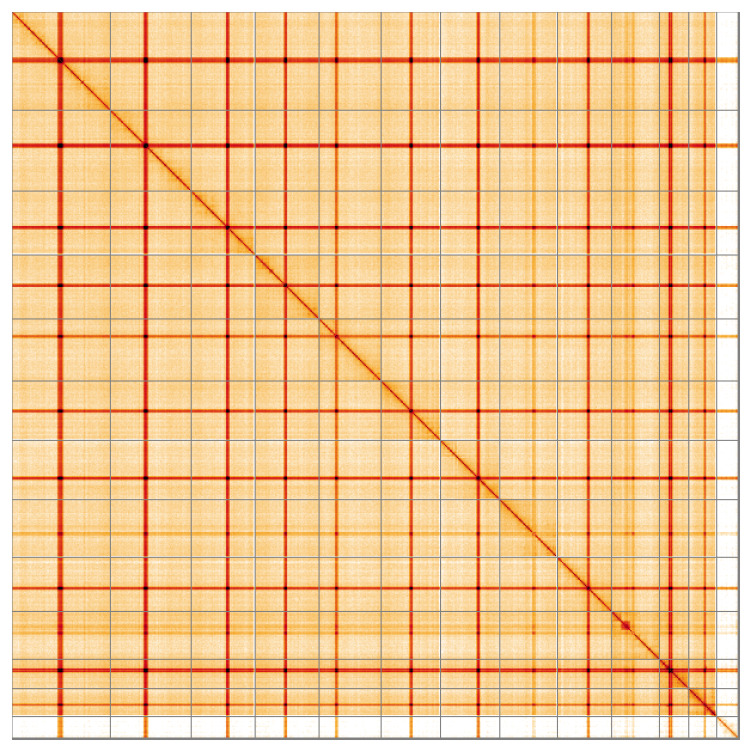
Genome assembly of *Xanthostigma xanthostigma* irXanXant1.1: Hi-C contact map of the irXanXant1.1 assembly, visualised using HiGlass. Chromosomes are shown in order of size from left to right and top to bottom. An interactive version of this figure may be viewed at https://genome-note-higlass.tol.sanger.ac.uk/l/?d=eRrgMcXATDqR-2U793m1Sg.

Most of the assembly sequence (99.74%) was assigned to 13 chromosomal-level scaffolds, representing 12 autosomes and the X sex chromosome. These chromosome-level scaffolds, confirmed by the Hi-C data, are named in order of size ( Figure 5; Table 3). 

**Table 3.** Chromosomal pseudomolecules in the genome assembly of *Xanthostigma xanthostigma*, irXanXant1.

INSDC accession	Name	Length (Mb)	GC%
OY754464.1	1	84.21	33.0
OY754465.1	2	69.27	33.0
OY754466.1	3	54.71	33.0
OY754467.1	4	54.71	33.5
OY754468.1	5	53.13	33.0
OY754469.1	6	50.87	33.0
OY754470.1	7	50.7	33.0
OY754471.1	8	49.42	33.0
OY754472.1	9	46.35	33.5
OY754473.1	10	40.92	34.0
OY754474.1	11	25.13	34.5
OY754475.1	12	23.97	34.0
OY754476.1	X	18.36	32.5
OY754477.1	MT	0.02	20.5

While not fully phased, the assembly deposited is of one haplotype. Contigs corresponding to the second haplotype have also been deposited. The mitochondrial genome was also assembled and can be found as a contig within the multifasta file of the genome submission, and as a separate fasta file with accession OY754477.1.

The primary assembly has a Quality Value (QV) of 66.1. The *k*-mer completeness of the primary assembly was estimated as 76.54%, the alternate haplotype 72.95% and the combined assemblies was 98.88%. BUSCO (v5.4.3) analysis using the endopterygota_odb10 reference set ( *n* = 2,124) indicated a completeness score of 97.9% (single = 96.8%, duplicated = 1.1%). The assembly achieves the EBP reference standard of 6.C.66. Other quality metrics are given in Table 2.

## Genome annotation report

The *Xanthostigma xanthostigma* genome assembly (GCA_963575645.1) was annotated at the European Bioinformatics Institute (EBI) on Ensembl Rapid Release. The resulting annotation includes 18,622 transcribed mRNAs from 13,251 protein-coding and 500 non-coding genes ( Table 2; https://rapid.ensembl.org/Xanthostigma_xanthostigma_GCA_963575645.1/Info/Index). The average transcript length is 9,412.19, with an average 1.35 coding transcripts per gene and 4.98 exons per transcript.

## Methods

### Sample acquisition and DNA barcoding

An adult male *Xanthostigma xanthostigma* (specimen ID Ox001530, ToLID irXanXant1) was collected from Wytham Woods, Berkshire, United Kingdom (latitude 51.76, longitude –1.33) on 2021-05-31 by netted. The specimen was collected by Steven Falk (independent researcher), identified by Liam Crowley (University of Oxford) and preserved on dry ice.

The specimen used for Hi-C sequencing (specimen ID NHMUK015073966, ToLID irXanXant3) was an adult specimen collected from Natural History Museum, South Kensington, England, United Kingdom (latitude 51.29, longitude –0.10) on 2022-07-28. The specimen was collected and identified by Maxwell Barclay (Natural History Museum) and preserved by dry freezing at –80 °C.

The initial identification was verified by an additional DNA barcoding process according to the framework developed by Twyford *et al*. (2024). A small sample was dissected from the specimens and stored in ethanol, while the remaining parts were shipped on dry ice to the Wellcome Sanger Institute (WSI). The tissue was lysed, the COI marker region was amplified by PCR, and amplicons were sequenced and compared to the BOLD database, confirming the species identification ( Crowley *et al.*, 2023). Following whole genome sequence generation, the relevant DNA barcode region was also used alongside the initial barcoding data for sample tracking at the WSI ( Twyford *et al.*, 2024). The standard operating procedures for Darwin Tree of Life barcoding have been deposited on protocols.io ( Beasley *et al.*, 2023).

### Nucleic acid extraction

The workflow for high molecular weight (HMW) DNA extraction at the WSI Tree of Life Core Laboratory includes a sequence of procedures: sample preparation and homogenisation, DNA extraction, fragmentation and purification. Detailed protocols are available on protocols.io ( Denton *et al.*, 2023b). The irXanXant1 sample was prepared for DNA extraction by weighing and dissecting it on dry ice ( Jay *et al.*, 2023). Tissue from the whole organism was homogenised using a PowerMasher II tissue disruptor ( Denton *et al.*, 2023a).

HMW DNA was extracted in the WSI Scientific Operations core using the Automated MagAttract v2 protocol ( Oatley *et al.*, 2023). The DNA was sheared into an average fragment size of 12–20 kb in a Megaruptor 3 system ( Bates *et al.*, 2023). Sheared DNA was purified by solid-phase reversible immobilisation, using AMPure PB beads to eliminate shorter fragments and concentrate the DNA ( Strickland *et al.*, 2023). The concentration of the sheared and purified DNA was assessed using a Nanodrop spectrophotometer and Qubit Fluorometer using the Qubit dsDNA High Sensitivity Assay kit. Fragment size distribution was evaluated by running the sample on the FemtoPulse system.

### Hi-C preparation

Tissue from the whole organism of the irXanXant3 sample was processed at the WSI Scientific Operations core, using the Arima-HiC v2 kit. Tissue (stored at –80 °C) was fixed, and the DNA crosslinked using a TC buffer with 22% formaldehyde. After crosslinking, the tissue was homogenised using the Diagnocine Power Masher-II and BioMasher-II tubes and pestles. Following the kit manufacturer's instructions, crosslinked DNA was digested using a restriction enzyme master mix. The 5’-overhangs were then filled in and labelled with biotinylated nucleotides and proximally ligated. An overnight incubation was carried out for enzymes to digest remaining proteins and for crosslinks to reverse. A clean up was performed with SPRIselect beads prior to library preparation.

### Library preparation and sequencing

Library preparation and sequencing were performed at the WSI Scientific Operations core. Libraries were prepared using the PacBio Express Template Preparation Kit v2.0 (Pacific Biosciences, California, USA) as per the manufacturer's instructions. The kit includes the reagents required for removal of single-strand overhangs, DNA damage repair, end repair/A-tailing, adapter ligation, and nuclease treatment. Library preparation also included a library purification step using AMPure PB beads (Pacific Biosciences, California, USA) and size selection step to remove templates shorter than 3 kb using AMPure PB modified SPRI. DNA concentration was quantified using the Qubit Fluorometer v2.0 (Thermo Fisher Scientific) and Qubit HS Assay Kit and the final library fragment size analysis was carried out using the Agilent Femto Pulse Automated Pulsed Field CE Instrument (Agilent Technologies). Samples were sequenced using the Sequel IIe system (Pacific Biosciences, California, USA). The concentration of the library loaded onto the Sequel IIe was in the range 40–135 pM. The SMRT link software, a PacBio web-based end-to-end workflow manager, was used to set-up and monitor the run, as well as perform primary and secondary analysis of the data upon completion.

For Hi-C library preparation, DNA was fragmented to a size of 400 to 600 bp using a Covaris E220 sonicator. The DNA was then enriched, barcoded, and amplified using the NEBNext Ultra II DNA Library Prep Kit following manufacturers’ instructions. The Hi-C sequencing was performed using paired-end sequencing with a read length of 150 bp on an Illumina NovaSeq 6000 instrument.

### Genome assembly, curation and evaluation

***Assembly***

The HiFi reads were first assembled using Hifiasm ( Cheng *et al.*, 2021) with the --primary option. Haplotypic duplications were identified and removed using purge_dups ( Guan *et al.*, 2020). The Hi-C reads were mapped to the primary contigs using bwa-mem2 ( Vasimuddin *et al.*, 2019). The contigs were further scaffolded using the provided Hi-C data ( Rao *et al.*, 2014) in YaHS ( Zhou *et al.*, 2023) using the --break option for handling potential misassemblies. The scaffolded assemblies were evaluated using Gfastats ( Formenti *et al.*, 2022), BUSCO ( Manni *et al.*, 2021) and MERQURY.FK ( Rhie *et al.*, 2020).

The mitochondrial genome was assembled using MitoHiFi ( Uliano-Silva *et al.*, 2023), which runs MitoFinder ( Allio *et al.*, 2020) and uses these annotations to select the final mitochondrial contig and to ensure the general quality of the sequence.

***Assembly curation***

The assembly was decontaminated using the Assembly Screen for Cobionts and Contaminants (ASCC) pipeline (article in preparation). Flat files and maps used in curation were generated in TreeVal ( Pointon *et al.*, 2023). Manual curation was primarily conducted using PretextView ( Harry, 2022), with additional insights provided by JBrowse2 ( Diesh *et al.*, 2023) and HiGlass ( Kerpedjiev *et al.*, 2018). Scaffolds were visually inspected and corrected as described by Howe *et al*. (2021). Any identified contamination, missed joins, and mis-joins were corrected, and duplicate sequences were tagged and removed. The sex chromosome was identified by read coverage statistics. The curation process is documented at https://gitlab.com/wtsi-grit/rapid-curation (article in preparation).

***Assembly quality assessment***

The Merqury.FK tool ( Rhie *et al.*, 2020) was used to evaluate *k*-mer completeness and assembly quality for the primary and alternate haplotype, using the *k*-mer databases ( *k* = 31) that were computed prior to genome assembly. The analysis outputs included assembly QV scores and completeness statistics.

A Hi-C contact map was produced for the final, public version of the assembly. The Hi-C reads were aligned using bwa-mem2 ( Vasimuddin *et al.*, 2019) and the alignment files were combined using SAMtools ( Danecek *et al.*, 2021). The Hi-C alignments were converted into a contact map using BEDTools ( Quinlan & Hall, 2010) and the Cooler tool suite ( Abdennur & Mirny, 2020). The contact map is visualised in HiGlass ( Kerpedjiev *et al.*, 2018).

The blobtoolkit pipeline is a Nextflow ( Di Tommaso *et al.*, 2017) port of the previous Snakemake Blobtoolkit pipeline ( Challis *et al.*, 2020). It aligns the PacBio reads in SAMtools and minimap2 ( Li, 2018) and generates coverage tracks for regions of fixed size. In parallel, it queries the GoaT database ( Challis *et al.*, 2023) to identify all matching BUSCO lineages to run BUSCO ( Manni *et al.*, 2021). For the three domain-level BUSCO lineages, the pipeline aligns the BUSCO genes to the UniProt Reference Proteomes database ( Bateman *et al.*, 2023) with DIAMOND blastp ( Buchfink *et al.*, 2021). The genome is also divided into chunks according to the density of the BUSCO genes from the closest taxonomic lineage, and each chunk is aligned to the UniProt Reference Proteomes database using DIAMOND blastx. Genome sequences without a hit are chunked using seqtk and aligned to the NT database with blastn ( Altschul *et al.*, 1990). The blobtools suite combines all these outputs into a blobdir for visualisation.

The blobtoolkit pipeline was developed using nf-core tooling ( Ewels *et al.*, 2020) and MultiQC ( Ewels *et al.*, 2016), relying on the Conda package manager, the Bioconda initiative ( Grüning *et al.*, 2018), the Biocontainers infrastructure ( da Veiga Leprevost *et al.*, 2017), as well as the Docker ( Merkel, 2014) and Singularity ( Kurtzer *et al.*, 2017) containerisation solutions.

Table 4 contains a list of relevant software tool versions and sources.

**Table 4.** Software tools: versions and sources.

Software tool	Version	Source
BEDTools	2.30.0	https://github.com/arq5x/bedtools2
BLAST	2.14.0	ftp://ftp.ncbi.nlm.nih.gov/blast/executables/blast+/
BlobToolKit	4.3.7	https://github.com/blobtoolkit/blobtoolkit
BUSCO	5.4.3 and 5.5.0	https://gitlab.com/ezlab/busco
bwa-mem2	2.2.1	https://github.com/bwa-mem2/bwa-mem2
Cooler	0.8.11	https://github.com/open2c/cooler
DIAMOND	2.1.8	https://github.com/bbuchfink/diamond
fasta_windows	0.2.4	https://github.com/tolkit/fasta_windows
FastK	427104ea91c78c3b8b8b49f1a7d6bbeaa869ba1c	https://github.com/thegenemyers/FASTK
Gfastats	1.3.6	https://github.com/vgl-hub/gfastats
GoaT CLI	0.2.5	https://github.com/genomehubs/goat-cli
Hifiasm	0.19.8-r587	https://github.com/chhylp123/hifiasm
HiGlass	44086069ee7d4d3f6f3f0012569789ec138f42b84 aa44357826c0b6753eb28de	https://github.com/higlass/higlass
Merqury.FK	d00d98157618f4e8d1a9190026b19b471055b22e	https://github.com/thegenemyers/MERQURY.FK
MitoHiFi	3	https://github.com/marcelauliano/MitoHiFi
MultiQC	1.14, 1.17, and 1.18	https://github.com/MultiQC/MultiQC
NCBI Datasets	15.12.0	https://github.com/ncbi/datasets
Nextflow	23.04.0-5857	https://github.com/nextflow-io/nextflow
PretextView	0.2.5	https://github.com/sanger-tol/PretextView
purge_dups	1.2.5	https://github.com/dfguan/purge_dups
samtools	1.16.1, 1.17, and 1.18	https://github.com/samtools/samtools
sanger-tol/ascc	-	https://github.com/sanger-tol/ascc
sanger-tol/blobtoolkit	0.6.0	https://github.com/sanger-tol/blobtoolkit
Seqtk	1.3	https://github.com/lh3/seqtk
Singularity	3.9.0	https://github.com/sylabs/singularity
TreeVal	1.0.0	https://github.com/sanger-tol/treeval
YaHS	1.2a.2	https://github.com/c-zhou/yahs

### Genome annotation

The Ensembl Genebuild annotation system ( Aken *et al.*, 2016) was used to generate annotation for the *Xanthostigma xanthostigma* assembly (GCA_963575645.1) in Ensembl Rapid Release at the EBI. Annotation was created primarily through alignment of transcriptomic data to the genome, with gap filling via protein-to-genome alignments of a select set of proteins from UniProt ( UniProt Consortium, 2019).

### Wellcome Sanger Institute – Legal and Governance

The materials that have contributed to this genome note have been supplied by a Darwin Tree of Life Partner. The submission of materials by a Darwin Tree of Life Partner is subject to the **‘Darwin Tree of Life Project Sampling Code of Practice’**, which can be found in full on the Darwin Tree of Life website here. By agreeing with and signing up to the Sampling Code of Practice, the Darwin Tree of Life Partner agrees they will meet the legal and ethical requirements and standards set out within this document in respect of all samples acquired for, and supplied to, the Darwin Tree of Life Project.

Further, the Wellcome Sanger Institute employs a process whereby due diligence is carried out proportionate to the nature of the materials themselves, and the circumstances under which they have been/are to be collected and provided for use. The purpose of this is to address and mitigate any potential legal and/or ethical implications of receipt and use of the materials as part of the research project, and to ensure that in doing so we align with best practice wherever possible. The overarching areas of consideration are:

•   Ethical review of provenance and sourcing of the material

•   Legality of collection, transfer and use (national and international)

Each transfer of samples is further undertaken according to a Research Collaboration Agreement or Material Transfer Agreement entered into by the Darwin Tree of Life Partner, Genome Research Limited (operating as the Wellcome Sanger Institute), and in some circumstances other Darwin Tree of Life collaborators.

## Data Availability

European Nucleotide Archive: Xanthostigma xanthostigma. Accession number PRJEB66774; https://identifiers.org/ena.embl/PRJEB66774. The genome sequence is released openly for reuse. The *Xanthostigma xanthostigma* genome sequencing initiative is part of the Darwin Tree of Life (DToL) project. All raw sequence data and the assembly have been deposited in INSDC databases. Raw data and assembly accession identifiers are reported in Table 1 and Table 2.

## Author information

Members of the University of Oxford and Wytham Woods Genome Acquisition Lab are listed here: https://doi.org/10.5281/zenodo.12157525.

Members of the Natural History Museum Genome Acquisition Lab are listed here: https://doi.org/10.5281/zenodo.12159242.

Members of the Darwin Tree of Life Barcoding collective are listed here: https://doi.org/10.5281/zenodo.12158331.

Members of the Wellcome Sanger Institute Tree of Life Management, Samples and Laboratory team are listed here: https://doi.org/10.5281/zenodo.12162482.

Members of Wellcome Sanger Institute Scientific Operations: Sequencing Operations are listed here: https://doi.org/10.5281/zenodo.12165051.

Members of the Wellcome Sanger Institute Tree of Life Core Informatics team are listed here: https://doi.org/10.5281/zenodo.12160324.

Members of the Tree of Life Core Informatics collective are listed here: https://doi.org/10.5281/zenodo.12205391.

Members of the Darwin Tree of Life Consortium are listed here: https://doi.org/10.5281/zenodo.4783558.
