# Supplementary material for: The genome sequence of a snakefly, Xanthostigma xanthostigma (Schummel, 1832)
Source: Wellcome Open Res. 2025 Feb 7;10:52. [Version 1] doi: 10.12688/wellcomeopenres.23674.1 (PMC11871431; doi:10.12688/wellcomeopenres.23674.1)
Supplement: Peer review history

# Reviewer report: Reviewer response for version 1

**DOI:** 10.21956/wellcomeopenres.26117.r118847

**Published:** 2025-03-12

**Authors:** Terrence Sylvester

**Affiliations:** 1 University of Memphis, Memphis, TN, USA

The authors present the genome sequence of the snakefly *Xanthostigma xanthostigma* and have done an excellent job assembling and scaffolding it to the chromosome level. They follow a standard, widely accepted genome sequencing and assembly pipeline, with well-presented and publicly available data. The methods are thoroughly detailed, including the programs used and their versions, ensuring the study's reproducibility. The assembly is of high quality, with a k-mer completeness of 98% and a quality value of 66.1. I have no major concerns regarding the work presented in this manuscript.

Are sufficient details of methods and materials provided to allow replication by others?

Yes

Is the rationale for creating the dataset(s) clearly described?

Yes

Are the datasets clearly presented in a useable and accessible format?

Yes

Are the protocols appropriate and is the work technically sound?

Yes

Reviewer Expertise:

Genomics

I confirm that I have read this submission and believe that I have an appropriate level of expertise to confirm that it is of an acceptable scientific standard.

# Reviewer report: Reviewer response for version 1

**DOI:** 10.21956/wellcomeopenres.26117.r118375

**Published:** 2025-02-28

**Authors:** Merly Escalona

**Affiliations:** 1 University of California Santa Cruz, Santa Cruz, California, USA

The authors described the new genomic resources for the snake fly *Xanthostigma xanthostigma*. The resources include a high-quality nuclear genome assembly, its genome annotation, and a mitochondrial genome assembly alongside the sequencing data generated to build them.

 The manuscript is well-written, and it includes the importance of the generated resources. Methods are precise, robust, and easy to follow. 

 My only comment is related to the mitochondrial assembly. There are no specific metrics describing the quality of the mitochondrial genome.

Are sufficient details of methods and materials provided to allow replication by others?

Yes

Is the rationale for creating the dataset(s) clearly described?

Yes

Are the datasets clearly presented in a useable and accessible format?

Yes

Are the protocols appropriate and is the work technically sound?

Yes

Reviewer Expertise:

Genome assembly

I confirm that I have read this submission and believe that I have an appropriate level of expertise to confirm that it is of an acceptable scientific standard.

# Reviewer report: Reviewer response for version 1

**DOI:** 10.21956/wellcomeopenres.26117.r118855

**Published:** 2025-02-26

**Authors:** Komal Kumar Bollepogu Raja

**Affiliations:** 1 Baylor College of Medicine, Houston, TX, USA

The article by Falk S and Crowley L et al. presents high quality genome sequence of snake fly, *Xanthostigma* *xanthostigma*. The background and ecology of this species has been well presented in the manuscript. The authors have also made a note that the sequence data has been generated from a single male fly and is arranged in 13 chromosomal pseudomolecules. The quality metrics of the assembly are impressive with BUSCO of 97.9% and a k-mer completeness of 98.88%. The methodology is robust. Protocols for extracting the DNA, bioinformatics pipelines and tools are all included in the manuscript for reproducibility. Overall, the manuscript is well written and presented and the data is useful for other researchers.

Are sufficient details of methods and materials provided to allow replication by others?

Yes

Is the rationale for creating the dataset(s) clearly described?

Yes

Are the datasets clearly presented in a useable and accessible format?

Yes

Are the protocols appropriate and is the work technically sound?

Yes

Reviewer Expertise:

Bioinformatics, Single cell omics, Genomics and Neurobiology

I confirm that I have read this submission and believe that I have an appropriate level of expertise to confirm that it is of an acceptable scientific standard.
